# The Association of Deep Vein Thrombosis With Cancer Treatment Modality: Chemotherapy or Surgery?

**DOI:** 10.5812/ircmj.14722

**Published:** 2014-09-05

**Authors:** Mitra Samare Fekri, Mahdie Khalily Zade, Shima Fatehi

**Affiliations:** 1 Physiology Research Center, Kerman University of Medical Sciences, Kerman, IR Iran; 2 Clinical Research Unit, Afzalipoor Hospital, Kerman University of Medical Sciences, Kerman, IR Iran

**Keywords:** Chemotherapy, Deep Vein Thrombosis, Malignancy, Surgery

## Abstract

**Background:**

Deep vein thrombosis (DVT) is a well-recognized complication in patients with cancer. Chemotherapy and cancer surgery increase the risk of DVT in these patients. There are a few reports about the prevalence of DVT in patients with cancer regarding different managing modalities.

**Objectives:**

This study aimed to assess the prevalence of DVT in patients with cancer, who were hospitalized in teaching hospitals, according to their treatment intervention.

**Patients and Methods:**

A cross-sectional retrospective study was conducted on 602 patients with cancer in Kerman, Iran, during years 2006-2007. Among the subjects, 301 had been operated and the rest had received chemotherapy. The prevalence of DVT was determined based on patients’ variables, cancer factors, and therapeutic modalities.

**Results:**

Totally, 349 subjects (58%) were male. DVT incidence was 19.9%, most of the cases were over 40 years of age (82.2%), and 21.2% of males and 18.2% of females had developed DVT. The prevalence of DVT in chemotherapy group was higher than that in surgery group (21.9% and 17.9%, respectively); however, this difference was statistically insignificant. DVT developed more frequently in lung cancer (42%) with small cell carcinoma being the most common pathologic finding (42.9%) in those with lung cancer (P = 0.0001).

**Conclusions:**

DVT occurs frequently in patients with malignancies. In this study, there was no association between DVT prevalence and age as well as sex; nonetheless, the prevalence was significantly higher in some sites and in patients with certain pathologies. Although DVT prevalence was higher in chemotherapy than in surgery, the difference was insignificant. Informing patients with cancer about symptoms of DVT and prophylactic interventions are warranted.

## 1. Background

Venous thromboembolism (VTE) is common among patients with cancer, particularly in those receiving cancer treatments. The increased risk of deep venous thrombosis (DVT) in patients with cancer appears to be due to the effect of malignancy on each component of the Virchow triad, namely, venous stasis, blood components imbalance, and vessel wall damage. Each factor ultimately contributes to the alteration of normal blood flow, thereby increasing thrombus formation (1). Moreover, this prothrombotic state may be further exacerbated by chemotherapy, hormone therapy, and surgery (2-4). 

Malignancy is one of the most common and important acquired risk factors for DVT and patients with active malignancy have a fourfold to sevenfold higher incidence of symptomatic VTE than the general population has (5, 6). DVT, which is most often a treatable condition, is the second leading cause of death in patients with malignancies and is associated with decreased survival rate. Furthermore, sequels of a DVT event such as pulmonary emboli, bleeding, post-thrombotic syndrome, or pulmonary hypertension can affect the patients with DVT. In addition, economic impact of DVT is considerable (1). 

On the other hand, increasing prevalence of DVT in hospitalized patients with cancer is alarming. For instance, a study showed the DVT prevalence rate of 1% as well as doubled rate of pulmonary emboli in seven years (7). Although more VTE cases are diagnosed nowadays by modern imaging techniques, surgical treatment or chemotherapy may also explain this growing trend (1).

## 2. Objectives

This study focuses on the prevalence rate of DVT in patients with cancer regarding the risk factors, malignancy location, pathologic findings, and the treatment strategy. 

## 3. Materials and Methods

This retrospective cross-sectional study was performed on 602 known cases of cancer who had been admitted in three major tertiary referral centers and teaching hospitals of Kerman City, Iran. The patients were recruited to this study by a simple nonrandomized sampling from October 2006 through August 2007. According to previous studies and with regard to maximum sample size for comparing two proportions, the sample size was calculated at 602 (301 subjects in each group). The statistical power was 80%. 

The study was based on the recorded data in medical files of patients with cancer in the archives of three teaching hospitals. We excluded all patients who had been treated with both surgery and chemotherapy as well as those who had received anticoagulant or antiplatelet drugs prior to treatment modalities. In this manner, 30 subjects in surgery group and 14 in chemotherapy group were excluded. Code of ethics was achieved and all patients’ information was kept confidential. The following data were recorded in researcher-designed checklist: demographic characteristics, medical history, site of tumor, pathology of cancers, and using Doppler sonography in diagnosis of DVT. Doppler sonography had been done for all patients with suspicious symptoms and signs of DVT by three calibrated instruments (Accuvix v10 A, Sony Corporation, Tokyo, Japan). The patients were allocated into two equal groups of chemotherapy and surgery. 

According to the number of patients who had DVT in the two groups, we calculated the prevalence of DVT and measured the association between the prevalence of DVT and patients’ variables as well as the treatment intervention. Patients’ data included their age, sex, underlying diseases such as chronic lung disease, congestive heart failure (CHF) and inactivity, oral contraceptive pills (OCP) consumption, hormone replacement therapy (HRT), and previous history of DVT. Chronic lung disease and CHF were determined according to patient's medication or signs and symptoms of lung disease asserted by the patient. Only patients who were taking OCP or HRT were considered as consumers of these medications. Inactivity was defined as being bedridden for at least three days. Cancer factors included the site, and histopathologic finding. Treatment data were the type of therapeutic modality, i.e. chemotherapy or surgery. One observer searched and recorded data from files. DVT diagnosis was made in the case of positive signs and symptoms including edema, pain, reddish-blue discoloration, and warming, especially when it was unilateral, and was confirmed with positive finding for DVT in Doppler ultrasonography. Only patients with positive findings in Doppler ultrasonography were enrolled in the study. 

Data collection and analysis were done with consideration of ethical issues. Data were analyzed by SPSS (v.16, SPSS Inc., Chicago, IL, USA). Independent variables for each group were summarized with descriptive analysis. Intergroup comparisons of demographic data, pathologic findings, cancer site, and treatment were performed with Chi square test. Fisher’s exact test was used for finding the correlation between risk factors and DVT. In all tests, P value < 0.05 was considered as statistically significant level.

## 4. Results

We recruited 602 patients including 349 males (56%). Total DVT prevalence rate was 19.9%; Although DVT was seen in 21.2% of males and 18.2% of females, there was no significant correlation between sex and DVT prevalence (P = 0.409). Patients with 40 to 65 and over 65 years of age (50% and 32.2% of the participants, respectively) had the highest DVT prevalence rate (21.6% and 20.1%, respectively); however, we could not find any statistically significant correlation between age and DVT prevalence (P = 0.459). The highest rate of DVT was seen consecutively in those with lung (42%), liver and biliary tract (25%), thyroid and pancreas (23.7%), urogenital system (20.6%), hematopoietic and bone marrow (9.6%), and breast cancers (13%) (P = 0.0001, Table 1). Small cell carcinoma was the pathologic finding that was associated with the highest DVT prevalence (42.9%). Adenocarcinoma, squamous cell carcinoma, other bronchogenic tumors, lymphoma, breast, and leukemia (26.9%, 23.1%) had the highest prevalence of DVT after that (P < 0.0001, Table 1). In our study, DVT affected all patients receiving OCP or HRT, inactive patients, or those with previous DVT. Overall, 76.1% of those who were affected by underlying diseases had DVT (P < 0.0001, Table 1). Although DVT prevalence rate was high in patients who had surgery (17.9%), the rate was higher in patients who received chemotherapy (21.9%) (P = 0.131).

**Table 1.** The Frequency of Deep Venous Thrombosis Based on Patients’ Variables, Cancer Factors, and Treatment Modality ^a,b^

	With Diagnosis of DVT Without Diagnosis of DVT		Total	OR (95% CI)	P Value
**Patient Related Factors**					
**Sex**				1.175 (0.774-1.782)	0.409
Male	74 (21.2)	275 (78.8)	349 (58)		
Female	46 (18.2)	207 (81.8)	253 (42)		
**Age, y**				0.904 (0.697-1.171)	0.459
< 19	4 (11.4)	31 (88.6)	35 (5.8)		
20-39	12 (16.7)	60 (83.3)	72 (12)		
40-64	65 (21.6)	236 (78.4)	301 (50)		
> 65	39 (20.1)	155 (79.9)	194 (32.2)		
**No Underlying Disease**	69 (12.8)	471 (82.2)	540 (100)		< 0.0001
**OCP Consumption**	2 (100)	0 (0)	2 (0.3)		< 0.0001
**HRP**	2 (100)	0 (0)	2 (0.3)		< 0.0001
**Obesity**	2 (100)	0 (0)	2 (0.3)		< 0.0001
**Physical Activity**	2 (100)	0 (0)	2 (0.3)		< 0.0001
**Previous DVT**	12 (100)	0 (0)	1 (2)		< 0.0001
**Cancer Related Factors**					
**Site of Cancer**				1.02 (0.963-1.092)	< 0.0001
Lung	34 (42)	47 (58)	81 (13.5)		
Gastrointestinal	36 (23.4)	118 (76.6)	154 (25.6)		
Liver and Biliary Tract	7 (25)	21 (75)	28 (4.7)		
Thyroid and Pancreas	9 (23.7)	29 (76.3)	38 (6.3)		
Urogenital System	13 (20.6)	50 (79.4)	63 (10.5)		
Hematopoietic	13 (9.6)	122 (90.4)	135 (22.4)		
Breast	6 (13)	40 (87)	46 (7.6)		
Mouth and Pharynx	17 (7.7)	12 (92.3)	13 (2.2)		
Skin	1 (3.2)	30 (96.8)	31 (5.1)		
Bone	0 (0)	13 (100)	13 (2.2)		
**Pathology**				1.03 (0.944-1.13)	< 0.0001
Small Cell Carcinoma	3 (42.5)	4 (57.1)	7 (1.2)		
Adenocarcinoma	58 (26.9)	158 (73.1)	216 (35.9)		
Squamous Cell Carcinoma	24 (23.1)	80 (76.9)	104 (17.2)		
Other Bronchogenic Tumors	5 (45.5)	6 (54.5)	11 (1.7)		
Leukemia	9 (9.2)	89 (90.8)	98 (16.3)		
Lymphoma	6 (14.3)	36 (85.7)	42 (6.9)		
Sarcoma	0 (0)	22 (100)	22 (3.7)		
Breast Carcinoma	6 (13.6)	39 (86.7)	45 (7.5)		
Others	9 (15.8)	48 (84.2)	57 (9.6)		
**Treatment related factors**					0.131
Chemotherapy	66 (21.9)	235 (78.1)	301 (50)		
Surgery	54 (17.9)	247 (82.1)	301 (50)		

^a^ Data are presented as No.(%).

^b^ Abbreviations: DVT, deep venous thrombosis; OR, odds ratio; OCP, oral contraceptive pills; and HRP, hormone replacement therapy.

## 5. Discussion

Most of the patients investigated in the present study were men (56%) and were over 40 years old. Total prevalence of DVT was 19.9% with more involvement of males in comparison with females. DVT was seen more frequently in the age group of 40 to 65 years than in other age groups. Although DVT was seen more in males and older people, the association between DVT and age as well as sex was insignificant. Patients with lung cancer had the highest frequency of DVT. DVT was identified more in some pathologic findings such as small cell carcinoma, adenocarcinoma, and squamous cell carcinoma, consecutively. In addition, this study showed the negative effect of OCP and HRP, inactivity, and history of previous DVT on developing DVT. Both surgery and chemotherapy were associated with increased risk of DVT; however, chemotherapy had stronger association with DVT. The prevalence of DVT in this study was 19.9% that was very higher than values reported in studies in Philippine (0.45%), the Netherlands (1.23%), and the United States (3.4%) (7-9). Another study estimated that up to 15% of patients with cancer develop clinically apparent DVT; nonetheless, these results might have underestimated the problem because according to postmortem studies, the prevalence of both symptomatic and undiagnosed DVT in advanced diseases is thought to be as high as 52% (10, 11). 

It is notable that our subjects were hospitalized patients for surgery, chemotherapy, or exacerbation of underlying diseases; therefore, they were not the representative of the whole population of patients with cancer, which can explain the higher prevalence rate of DVT in this study in comparison with some others researches. 

Although the risk of DVT increased with age, there was not any correlation between age and the prevalence of DVT in our study. In many studies, age was an independent risk factor for VTE in hospitalized patients with cancer (2, 4, 6); however, advanced age was not an important risk factor in some other retrospective studies (3, 12, 13). This difference might be due to different sample size for estimation of this association or delayed cancer diagnosis in some of those studies. The increasing rate of DVT with age might be due to the increasing rate of DVT risk factors such as hypertension, chronic renal disease, chronic liver disease, CHF, and inactivity with age or difference in pathologic type or location of cancer in different age groups. 

We could not find any correlation between sex and prevalence of DVT, but DVT was seen more frequently in males. There are contrary findings in literature in this regard; whilst most studies have reported higher frequency of DVT in males in comparison to females, a newer pooled study has found the opposite result and some studies have reported equal sex ratio (3, 14-16). 

In this study, the prevalence of DVT was higher (16.8%) in patients with solid cancers than in those with hematologic malignancies (3, 17). Some other studies obtained similar prevalence in patients with solid and hematologic cancers (28). In our study, patients with hematologic cancer were younger or had less underlying diseases in comparison with those with solid cancers. In the present study, DVT prevalence showed difference based on site of tumor; the highest proportion of DVT cases was seen in the lung cancer followed by liver and biliary tract, thyroid, pancreas, GI tract, and genitourinary tract cancers, consecutively. This finding is almost different from others; for example, in one study cancers of pancreas, other abdominal tumors, ovary, and kidney tumors had the highest risk of DVT development, consecutively (7). In another investigation on hospitalized patients, the most prevalent tumors were pancreas tumors, lymphoma, and brain malignancies (2). Whilst some researchers believe that pancreas cancer is associated with the highest prevalence of DVT (3, 7), according to available studies, lung cancer has the high prevalence of DVT, especially during chemotherapy (7.3-24%). Although lung cancer does not have the highest prevalence among all cancers (1, 16, 17), it had the highest prevalence in some studies (8, 9). DVT prevalence increases with increasing the tumor stage, cancer chemotherapy, and non-small cell carcinoma (18). In one study on Arab patients with cancer, the most common thrombosis-associated malignancies were consecutively breast, non-Hodgkin’s lymphoma, and lung cancer and majority of DVT cases had developed in advanced stages of disease (7). According to a large study on Japanese patients with cancer who underwent chemotherapy, stomach, pancreas, and lung cancers were associated with very high risk of DVT (19.2%, 15.8%, and 13.9%, respectively) (5). In addition, a study on American patients with cancer determined the metastatic stage of pancreatic cancer as a leading cause of thrombosis due to malignancies with developing DVT in 20% of patients in a one-year follow-up, whilst 5% of patients with lung cancer had developed thrombosis (15). Lung, as the most common site for DVT occurrence, might be a dependent variable in our study since cigarette smoking or opium addiction is higher in lung patients with cancer than in other patients with cancer, which puts this group at risk of complications such as atherosclerosis or chronic obstructive pulmonary disease that predispose these patients to thrombosis. We did not have any patient with malignant brain tumor; therefore, we could not find thrombosis risk of this cancer. 

DVT prevalence was different based on various pathologic findings. Small cell carcinoma, adenocarcinoma, and squamous cell carcinoma were the malignancies with highest rate of DVT consecutively whilst we did not found DVT in patients with bone sarcoma. Similar to our study, several studies have reported stronger association of DVT with adenocarcinoma than with squamous cell carcinoma (16, 19). In present study, the most prevalent pathology associated with DVT was small cell carcinoma of lung. It can be explained by high prevalence of lung cancer and its correlation with smoking. Furthermore, patients with small cell carcinoma show more aggressive course of disease so that most of them have metastasis and more severe illness at the time of diagnosis than other lung cancers have. 

We found higher prevalence of DVT in lymphoma than in leukemia, which was similar to the findings of an Italian study (20). In this study, DVT prevalence was correlated with some demographic features of patients. Low percentage of patients had developed DVT without any risk factor whilst DVT was found in the most patients with risk factors such as hypertension, OCP consumption, inactivity, previous DVT, chronic liver disease, and chronic heart disease. In our study, all patients with hypertension (2 patients), OCP consumption (2 patients), inactivity (2 patients), and previous DVT (12 patients), and with CHF or chronic lung disease (35 patients) developed DVT. There is an established association between abovementioned risk factors and DVT according to some researches (13, 17, 21). 

Although there was a relationship between chemotherapy and surgery with DVT formation, DVT was more frequent in patients who had undergone chemotherapy. In our study, both chemotherapy and surgery had the high risk of DVT development, even though DVT was insignificantly the more prevalent in patients with chemotherapy. This difference might be explained by sample size; however, type of surgery, whether it was major or minor, site of surgery, neglecting anticoagulant usage in surgery group, and underlying condition in both groups were factors that could affect this conclusion. Interestingly, we could not find a similar study comparing surgery and chemotherapy to determine which one would have a stronger associated with DVT consequence. In other studies, overall DVT prevalence rate has been reported to be between zero and 43% according to the kind of chemotherapy (1, 7). On the other hand, risk of DVT in patients with cancer who underwent surgery has been reported to be between zero and 52% in different studies (7, 10, 20). Several studies have shown the higher risk of chemotherapy in comparison with surgery in which the prevalence rate of DVT has reported to be about twofold as high as that of surgery (2, 17, 18). On the other hand, release procoagulant factors from damaged tumor cells after chemotherapy and inadequate production of anticoagulants factors might predispose patients to DVT. 

In addition to the large sample size that was a strong point of our study, we could not find any study with such a design in hospitalized patients with cancer, which highlights the importance of chemotherapy in DVT occurrence in comparison to the surgery. Nonetheless, since the DVT prevalence rate was high in both groups and with regard to increasing mortality and morbidity in these groups, a revised guideline to start early anticoagulation is seem necessary in hospitalized patients with cancer who are supposed to either undergo surgery or receive chemotherapy, especially in those with certain underlying diseases, cancer site, or pathologic findings. 

### 5.1. Study limitation

One of the limitations to this study was its retrospective design. In addition, other factors might play a role in thrombosis that we did not know or could not estimate their effect. Further well-designed perspective studies are required for confirming the effect of some of these risk factors.
